# Supplementary material for: Mechanisms governing the pioneering and redistribution capabilities of the non-classical pioneer PU.1
Source: Nat Commun. 2020 Jan 21;11:402. doi: 10.1038/s41467-019-13960-2 (PMC6972792; doi:10.1038/s41467-019-13960-2)
Supplement: Supplementary file 7 — Source data [file 41467_2019_13960_MOESM7_ESM.zip › Source_Data/FigureS2/FigureS2B_MotifScanOutput/KmeansCluster13/homerResults/motif33.similar.html]

motif33

## Information for motif33

A
C
T
G
A
T
G
C
C
G
A
T
A
G
C
T
C
G
T
A
C
G
T
A
C
T
G
A
C
A
G
T
C
T
A
G
A
G
T
C
C
G
T
A
C
T
G
A
C
G
T
A
A
C
G
T
  
Reverse Opposite:  

C
G
T
A
A
C
G
T
A
G
C
T
A
C
G
T
A
C
T
G
A
G
T
C
G
C
T
A
A
G
C
T
C
G
A
T
A
C
G
T
C
T
G
A
C
G
T
A
T
A
C
G
A
G
T
C
  

|  |  |
| --- | --- |
| p-value: | 1e-18 |
| log p-value: | -4.181e+01 |
| Information Content per bp: | 1.854 |
| Number of Target Sequences with motif | 14.0 |
| Percentage of Target Sequences with motif | 0.83% |
| Number of Background Sequences with motif | 2.8 |
| Percentage of Background Sequences with motif | 0.01% |
| Average Position of motif in Targets | 109.2 +/- 54.3bp |
| Average Position of motif in Background | 61.4 +/- 12.7bp |
| Strand Bias (log2 ratio + to - strand density) | -0.4 |
| Multiplicity (# of sites on avg that occur together) | 1.00 |
| Motif File: | file (matrix) reverse opposite |

### Similar de novo motifs found

|  |  |  |  |  |  |  |  |
| --- | --- | --- | --- | --- | --- | --- | --- |
| Rank | Match Score | Redundant Motif | P-value | log P-value | % of Targets | % of Background | Motif file |
| 1 | 0.864 | A C G T A C G T A C T G G T A C C G T A A G C T A C G T C G A T C G T A C G T A A T C G A G T C | 1e-13 | -30.752048 | 0.65% | 0.01% | motif file (matrix) |
| 2 | 0.876 | A C G T A G C T C G T A C G T A C T G A A C G T C T A G A G T C C G T A C G T A C G T A A C G T C T G A | 1e-12 | -29.100069 | 0.59% | 0.01% | motif file (matrix) |
